# The “Iron Gate” Outcompetes the “Enzymic Latch” as the Dominant Soil Organic Carbon Stabilization Mechanism in Permafrost Peatlands of the Great Hing’an Mountains

**DOI:** 10.3390/biology14111504

**Published:** 2025-10-28

**Authors:** Shuping Kan, Weiping Yin, Zhao Li, Xinmiao Guo, Dalong Ma, Huan Yu, Yiting Zhao

**Affiliations:** 1 College of Geographical Sciences, Harbin Normal University, Harbin 150025, China; 2024300998@stu.hrbnu.edu.cn (S.K.); 2024400071@stu.hrbnu.edu.cn (W.Y.); guoxinmiao2003@163.com (X.G.); yuhuan10729@163.com (H.Y.); 19846295607@163.com (Y.Z.); 2 Heilongjiang Wuyiling Wetland Ecosystem National Observation and Research Station, Yichun 153000, China; 3 Institute of Industrial Crops, Heilongjiang Academy of Agricultural Sciences, Harbin 150086, China

**Keywords:** permafrost peatlands, soil organic carbon, phenol oxidase, ferric iron, iron-bound soil organic carbon

## Abstract

**Simple Summary:**

The “enzymic latch” and “iron gate” theories represent two prevailing and contrasting mechanisms governing ecosystem carbon stability: the former via a phenolics accumulation mediated biochemical cascade that suppresses hydrolytic enzyme activity, and the latter via an abiotic pathway where ferrous iron oxidation suppresses phenol oxidase activity and promotes iron-bound soil organic carbon formation. Therefore, deciphering the stabilization mechanisms for the vast carbon stocks in permafrost peatlands represents a central challenge for climate change projections. In this study, we assessed the spatial distribution and interrelationships of peatland soil extracellular enzyme activities, iron phases, and iron-bound soil organic carbon across three permafrost zones in the Great Hing’an Mountains. Contrary to the “enzymic latch” mechanism, our data revealed that hydrolytic enzyme activities (β-glucosidase, cellobiohydrolase, and β-*N*-acetylglucosaminidase) were neither negatively correlated with phenolics nor positively correlated with phenol oxidase activity. Instead, iron emerged as the central regulator, with a positive correlation between ferrous iron and phenol oxidase activity and with ferric iron stabilizing soil organic carbon through co-precipitation. Our results highlighted that permafrost degradation could poses a threat to the dominant “iron gate” carbon sequestration mechanism in peatlands, potentially triggering a positive climate feedback.

**Abstract:**

Distinct paradigms, such as the “enzymic latch” and “iron gate” theories, have been proposed to elucidate SOC loss or accumulation, but their relative significance and whether they are mutually exclusive in permafrost peatlands remain unclear. To address this, we evaluated their relative importance and identified the dominant factors controlling SOC stability. Therefore, we employed a space-for-time substitution approach across a permafrost gradient (continuous, discontinuous, and isolated) by systematically quantifying extracellular enzyme activities, iron (Fe) phases, and iron-bound soil organic carbon (Fe-SOC) at various depths (0–10, 10–30, and 30–50 cm) in peatlands. Our results did not support the “enzymic latch” theory, with hydrolytic enzyme activities (β-glucosidase (BG), cellobiohydrolase (CBH), and β-*N*-acetylglucosaminidase (NAG)) showing positive correlations with phenolics but negative correlations with phenol oxidase (PHO) activity. However, ferrous iron (Fe(II)) was significantly positively correlated with PHO activity, and ferric iron (Fe(III)) stabilized SOC through co-precipitation with it to form Fe-SOC, supporting the “iron gate” theory. Moreover, Fe-SOC decreased from the continuous to the isolated permafrost zone, and with soil depth from 0–10 cm to 30–50 cm. Partial least squares path modeling (PLS-PM) analysis indicated that Fe(III) directly and indirectly (via Fe-SOC and phenolics) affected SOC. Our study demonstrated the primacy of the “iron gate” mechanism in controlling carbon stability in the Great Hing’an Mountains permafrost peatlands, providing new insights for projecting carbon-climate feedback.

## 1. Introduction

Peatlands occupy just 2.84% of the land area, but with their storage of approximately 30% of the world’s soil carbon, peatlands are crucial to the feedback between the climate change and global carbon cycle [1]. The long-term waterlogging, low temperatures and oxygen deficiency limit microbial metabolic activity and diminish the rates of organic matter decomposition, thereby making peatlands one of the most effective natural barriers against climate change [2]. The majority of peatlands are located in the middle and high latitudes of the Northern Hemisphere with over half being underlain by permafrost [3]. However, temperatures in high-latitude regions has risen to be two or three times of the global average, resulting in the accelerated thawing of permafrost where a large amount of sequestered carbon has been decomposed by microbial communities, releasing carbon dioxide (CO_2_) or methane (CH_4_) into the atmosphere, which potentially transforms permafrost peatlands from carbon sinks to sources [4,5]. Therefore, a comprehensive exploration of carbon stabilization mechanisms and its driving factors may improve our predictions of permafrost peatland carbon trajectories under the context of global change.

Hydrolytic and oxidative enzymes, the two main enzymatic classes of soil extracellular enzymes, play pivotal roles in the degradation and transformation of soil organic carbon (SOC) and are primarily produced by soil microorganisms [6]. Currently, two well-known mechanisms, the “enzymic latch” and “iron gate”, have been proposed to explain loss or accumulation of SOC in peatlands [7]. The “enzymic latch” theory suggests that the reduced phenol oxidase (PHO) activity results in the accumulation of phenolics which are toxic to hydrolytic enzymes, thereby reducing hydrolytic enzyme activities and preventing SOC decomposition [8]. During peatland water table drawdown, oxygen exposure breaks the “enzymic latch”, which in turn promotes the biodegradation of organic compounds [9]. However, several studies have reported conflicting evidence for the “enzymic latch” theory or indicated that the theory may not fully realize in some contexts [10,11,12]. This suggests that other confounding or competing mechanisms may regulate SOC dynamics in peatland ecosystems. Contrary to “enzymic latch”, the “iron gate” theory states ferrous iron (Fe(II)) as the key regulator of PHO activity, whereby the oxidation of Fe(II) to ferric iron (Fe(III)) suppresses PHO activity, thus counteracting the “enzymic latch” and promoting SOC conservation [13]. Furthermore, Fe and SOC can form iron-bound soil organic carbon (Fe-SOC) through adsorption and co-precipitation for stability of SOC in long-term [14]. Recent studies have increasingly highlighted on the role of Fe-SOC in soil carbon sequestration, with estimates indicating it stabilizes 37.8%, 15.8%, and 5.4–11.8% of SOC in forest, grassland, and wetland, respectively [15,16,17]. These two mechanisms provide crucial knowledge for knowing the potential carbon dynamics in peatlands that contain vast carbon stocks. Thus, understanding the relationship among extracellular enzymes, Fe phases, and Fe-SOC, along with the factors influencing them, will reduce existing uncertainties in quantifying carbon budgets and fluxes in peatland ecosystems, leading to a more accurate projection of carbon feedback due to permafrost degradation.

Located at the southernmost boundary of the Eurasian permafrost zone, the permafrost peatlands in the Great Hing’an Mountains serve as both an important indicator of global climate change and a pivotal component of carbon-water cycling in the Northern Hemisphere’s high latitudes [18]. Climate warming has triggered the thawing of permafrost, which subsequently results in a deeper soil active layer, changes in surface vegetation composition, a declining water table, altered carbon sequestration, and variations in microbial communities involved in the decomposition or transformation of organic carbon [19,20]. The thawing of high-latitude permafrost releases substantial organic carbon, posing an emerging issue of global concern [21]. Increasing evidence suggests that greenhouse gas emissions from peatlands are predicted to rise under future climate warming, while the mechanisms driving enhanced SOC decomposition remain poorly understood [22,23]. Despite extensive research on the “enzymic latch” and “iron gate” mechanisms, their potential interaction (co-existence or competition) in organic-rich permafrost peatlands remains unclear, and it is even less certain which mechanism will dominate in the context of permafrost degradation. In order to address these knowledge gaps, we employed a space-for-time substitution approach across different permafrost zones (continuous permafrost zone, discontinuous permafrost zone, and isolated permafrost zone) by examining extracellular enzyme activities, Fe phases, and Fe-SOC at various depths (0–10 cm, 10–30 cm, and 30–50 cm) in peatlands. The main objectives of this study were as follows: (1) to explore whether PHO is a key regulator for SOC content, (2) to determine whether Fe(III) plays a protective role in SOC along soil profiles, and (3) to clarify how the “enzymic latch” or “iron gate” mechanism mediate SOC storage in permafrost peatlands.

## 2. Materials and Methods

### 2.1. Study Area and Sample Collection

The research site is situated in the high-latitude permafrost region of the Great Hing’an Mountains (40°59′–53°33′ N, 115°05′–125°16′ E) in Northeastern China. Characterized by a typical cold temperate continental monsoon climate, this area experiences prolonged, severe winters and brief summers, with a mean annual temperature variation between −5.3 °C and −1.4 °C and distinct seasonal variations [24]. The Great Hing’an Mountains comprise three main permafrost zones: continuous permafrost zone (>90% areal permafrost coverage, CP), discontinuous permafrost zone (50–90% areal permafrost coverage, DP), and isolated permafrost zone (<10% areal permafrost coverage, IP) [25]. Peatlands in this area form and evolve over underlying permafrost, with the predominant vegetation including *Vaccinium uliginosum*, *Rhododendron tomentosum*, *Carex appendiculata*, and *Sphagnum palustre*. We selected nine typical peatland sampling sites across three permafrost gradients in September 2024, including Mohe, Tuqiang and Huzhong (CP1, CP2 and CP3) (1) situated in the continuous permafrost zone; Tahe, Xinlin and Hanma (DP1, DP2 and DP3) (2) situated in the discontinuous permafrost zone; and Songling, Jiagedaqi and Dayangshu (IP1, IP2 and IP3) (3) situated in the isolated permafrost zone. The thickness of active layer (60–115 cm) varied significantly across different permafrost gradients. Meanwhile, three independent 10 × 10 m quadrats were randomly established in each sampling site, and five replicate soil samples were collected from each at various depths (0–10 cm, 10–30 cm, and 30–50 cm). The samples from each depth were thoroughly mixed to generate composite samples, resulting in 81 soil samples (9 peatlands × 3 quadrats × 3 depths). Soil samples were delivered to the lab at 4 °C in sterile bags and subsequently removed roots, litter, and stones. One portion of the collected samples was air-dried for the analysis of soil physicochemical properties, and the other portion was preserved at 4 °C for the measurement of extracellular enzyme activities and Fe phases.

### 2.2. Analysis of Soil Physicochemical Properties

An elemental analyzer (Isoprime 100, Elementar, Langenselbold, Germany) was employed to measure the soil organic carbon (SOC) and total nitrogen (TN) contents. Dissolved organic carbon (DOC) was determined using a multi-N/C 3100 analyzer (Analytik Jena, Germany). The measurement of total phosphorus (TP) was conducted via the molybdenum blue method [26]. Soil water content (SWC) was measured by oven-drying soil samples at 105 °C for 24 h and calculated the resultant weight loss [27]. The pH of the soil was determined using a pH meter (LeiCi PHSJ-3F, Shanghai, China). Phenolics were measured using the Folin-Ciocalteau method [28].

### 2.3. Assay of Soil Extracellular Enzyme Activities

The activities of five hydrolytic enzymes (β-glucosidase (BG), cellobiohydrolase (CBH), β-*N*-acetylglucosaminidase (NAG), leucine aminopeptidase (LAP) and acid phosphatase (AP)) and two oxidative enzymes (phenol oxidase (PHO) and peroxidase (PER)) were determined using the method developed by Saiya-Cork et al. [29]. Methylumbelliferone (MUB) and L-dihydroxyphenylalanine (DOPA) were used as substrates to measure hydrolytic and oxidative enzyme activities, respectively. Soil sample suspensions were prepared by homogenizing 1 g of soil in 125 mL of 50 mM acetate buffer (pH 5.0) for 5 min. Then, this suspension (200 μL) along with 50 μL of substrate were transferred into 96-well microplates. Following 4 h of incubation at 20 °C in darkness, fluorescence was carried out using a microplate reader (BioTek Instruments, Swindon, UK) using an excitation wavelength of 365 nm and an emission detection wavelength of 450 nm. The measurement of PHO activity was performed by introducing 200 μL soil suspension and 50 μL DOPA solution into each microplate well. For PER assays, 10 μL of 0.3% H_2_O_2_ was additionally included. Following 18 hours of dark incubation at 20 °C, enzyme activities were determined by measuring the absorbance at 450 nm. Each assay included eight replicate wells per sample.

### 2.4. Measurement of Fe and Fe-SOC

Various Fe fractions (Fe(II), total Fe (Fe_t_) and free Fe oxide (Fe_d_)) were determined using the 1,10-Phenanthroline spectrophotometric method, with extractions performed using a 0.5 M HCl solution, concentrated hydrochloric acid solution, and a citrate-bicarbonate-dithionite (CBD) solution, respectively [30].

Fe(III) = Fe_t_ − Fe(II) (1)

Iron-bound soil organic carbon (Fe-SOC) was quantified following Lalonde et al. [31]. Soil sample extraction was performed using sodium chloride (NaCl) that had an equivalent ionic strength to CBD. Prior to drying, the remaining soil was thoroughly rinsed three times using 1 M NaCl. Following SOC determination, the Fe-SOC, C:Fe molar ratio and the proportion of Fe-SOC in SOC (f_Fe-SOC_) were computed using the following equations:

Fe-SOC = SOC_NaCl_ − SOC_CBD_ (2)

C:Fe molar ratio = (Fe-SOC/MC)/(Fe_d_/MFe) (3)

f_Fe-SOC_ = Fe-SOC/SOC × 100% (4)

SOC_NaCl_ and SOC_CBD_ represent the residual SOC fractions following NaCl and CBD extraction treatments, respectively. The molar masses of carbon and Fe are denoted as MC and MFe, respectively.

### 2.5. Statistical Analysis

Differences in soil physicochemical properties, extracellular enzyme activities and Fe phases of different permafrost peatlands were assessed via one-way ANOVA with SPSS 26.0, using a significance level of *p* < 0.05. Two-way ANOVA was used to determine the effects of soil depth, peatland type, and their interactions on soil extracellular enzyme activities and Fe and C coupling. The Kolmogorov–Smirnov (K-S) and Levene tests were used to assess normality and homogeneity of variance, respectively, and data transformations were applied when these assumptions were violated. Pearson correlations were used to visualize correlations between soil physicochemical properties, extracellular enzyme activities and Fe phases in a heatmap. The relationships between Fe(II) and PHO activity, between Fe(II) and BG activity, between PHO activity and pH, between BG activity and pH, between Fe(III) and SOC, and between Fe(III) and Fe-SOC were assessed based on linear correlation analysis. The “plspm” package in R (v.4.1.0) was employed to implement partial least squares path modeling (PLS-PM), evaluating the influences of soil physicochemical properties, extracellular enzyme activities, and Fe phases on SOC. Model quality was assessed through the goodness-of-fit (GOF) index.

## 3. Results

### 3.1. Soil Physicochemical Properties

The contents of SOC and DOC exhibited a declining trend with increasing depth. The SOC and DOC contents at 30–50 cm were significantly lower than at 0–10 cm in all peatlands (*p* < 0.05, Table 1). IP showed significantly lower SOC (at 0–10 cm and 10–30 cm) and DOC (at all depths) contents than CP (*p* < 0.05). Permafrost degradation may disrupt the stability of the legacy carbon pool and accelerate its mobilization and release processes. The content of TN in IP was significantly higher than in CP and DP at both 0–10 cm and 30–50 cm (*p* < 0.05). The TP content at 0–10 cm was significantly higher than that at other depths in all peatlands, except for in DP1 (*p* < 0.05). The content of TP in IP was significantly lower than in CP (*p* < 0.05). The divergent spatial distributions of nitrogen and phosphorus across permafrost zones indicate that degradation will fundamentally reconfigure nutrient regimes in peatland ecosystems. CP and DP showed significantly higher SWC than IP (*p* < 0.05), indicating that a deepening active layer facilitates rapid water loss. The soil pH exhibited a variation between 4.40 and 5.65 and was significantly higher in CP compared to other permafrost zones (*p* < 0.05). The phenolics content in IP was markedly higher than in CP at all depths (*p* < 0.05).

### 3.2. Soil Hydrolytic Enzyme Activities

**Figure 1:**
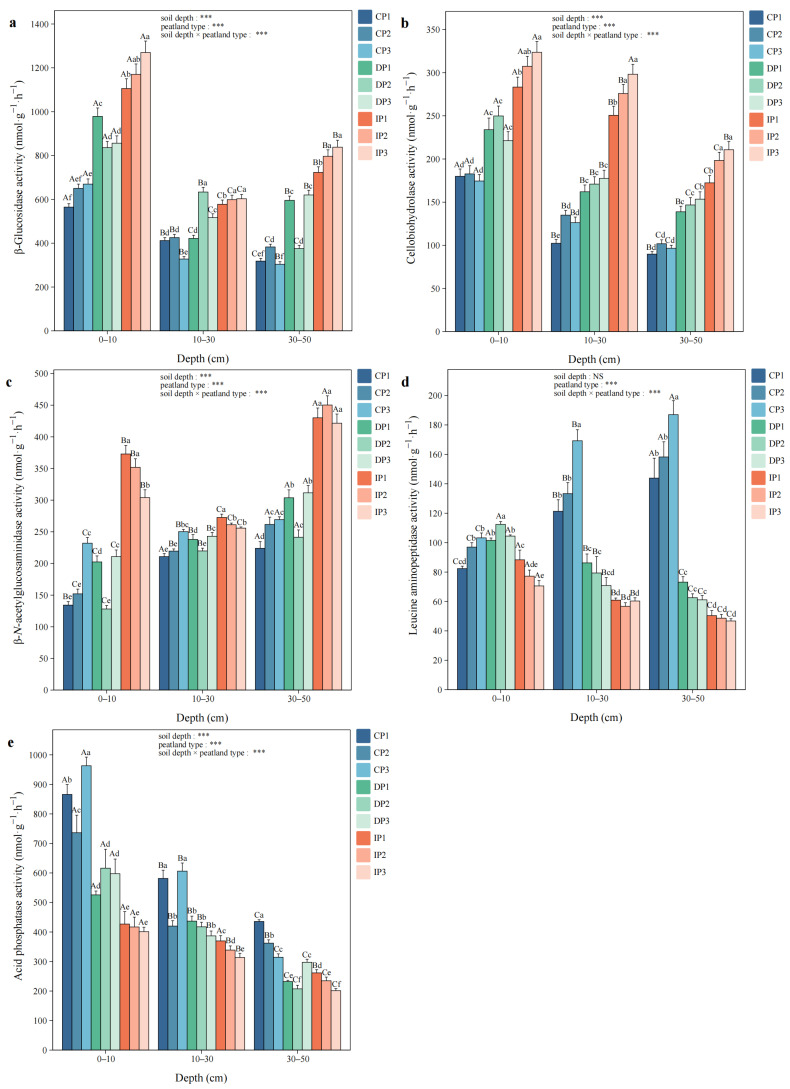
The activities of (**a**) β-glucosidase (BG), (**b**) cellobiohydrolase (CBH), (**c**) β-*N*-acetylglucosaminidase (NAG), (**d**) leucine aminopeptidase (LAP), and (**e**) acid phosphatase (AP). Capital letters indicate significant differences at different soil depths in the same peatland (*p* < 0.05), and lowercase letters indicate significant differences among different peatlands at the same depth (*p* < 0.05). Two-way ANOVA was conducted to determine the effects of soil depth, peatland type, and their interactions on soil hydrolytic enzyme activities (*** *p* < 0.001; NS, non-significant).

BG had the highest activity among all soil hydrolytic enzymes, with a variation range of 303.98–1269.77 nmol·g^−1^·h^−1^. Across the permafrost gradient (CP to IP), CBH activity exhibited an increasing trend at all soil depths. Both BG and CBH activities in IP were significantly higher than those in other permafrost zones, except for BG in DP2 at 10–30 cm and CBH in DP3 at 30–50 cm (*p* < 0.05). Furthermore, BG and CBH activities at 0–10 cm were significantly higher than those at other depths in all peatlands, except for CBH in IP3 at 10–30 cm (*p* < 0.05, Figure 1a,b). At 0–10 cm and 30–50 cm, NAG activity in IP was significantly higher compared to other permafrost zones (*p* < 0.05). Moreover, NAG activity at 30–50 cm was markedly higher than that at other depths, except for in CP1 at 10–30 cm (*p* < 0.05, Figure 1c). CP exhibited markedly higher LAP activity at 10–30 cm and 30–50 cm, while at 0–10 cm, LAP activity in IP was significantly lower than in DP (*p* < 0.05, Figure 1d). AP activity in CP was significantly higher than that in IP at all depths (*p* < 0.05, Figure 1e).

### 3.3. Soil Oxidative Enzyme Activities

**Figure 2:**
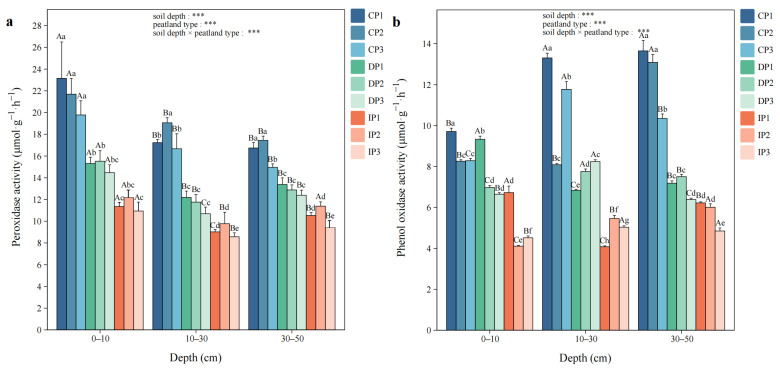
The activities of (**a**) peroxidase (PER) and (**b**) phenol oxidase (PHO). Capital letters indicate significant differences at different soil depths in the same peatland (*p* < 0.05), and lowercase letters indicate significant differences among different peatlands at the same depth (*p* < 0.05). Two-way ANOVA was conducted to determine the effects of soil depth, peatland type, and their interactions on soil oxidative enzyme activities (*** *p* < 0.001).

PER activity showed a decreasing trend from CP to IP and was significantly higher in CP at all soil depths compared to other permafrost zones (*p* < 0.05). Furthermore, PER activity was significantly higher at 0–10 cm than at 10–30 cm in all peatlands (*p* < 0.05, Figure 2a). PHO activity varied from 4.11 to 13.65 μmol·g^−1^·h^−1^ and the highest value occurred at 30–50 cm in CP1. Compared to IP, CP exhibited significantly higher PHO activity at all depths (*p* < 0.05, Figure 2b).

### 3.4. Fe and C Coupling

**Figure 3:**
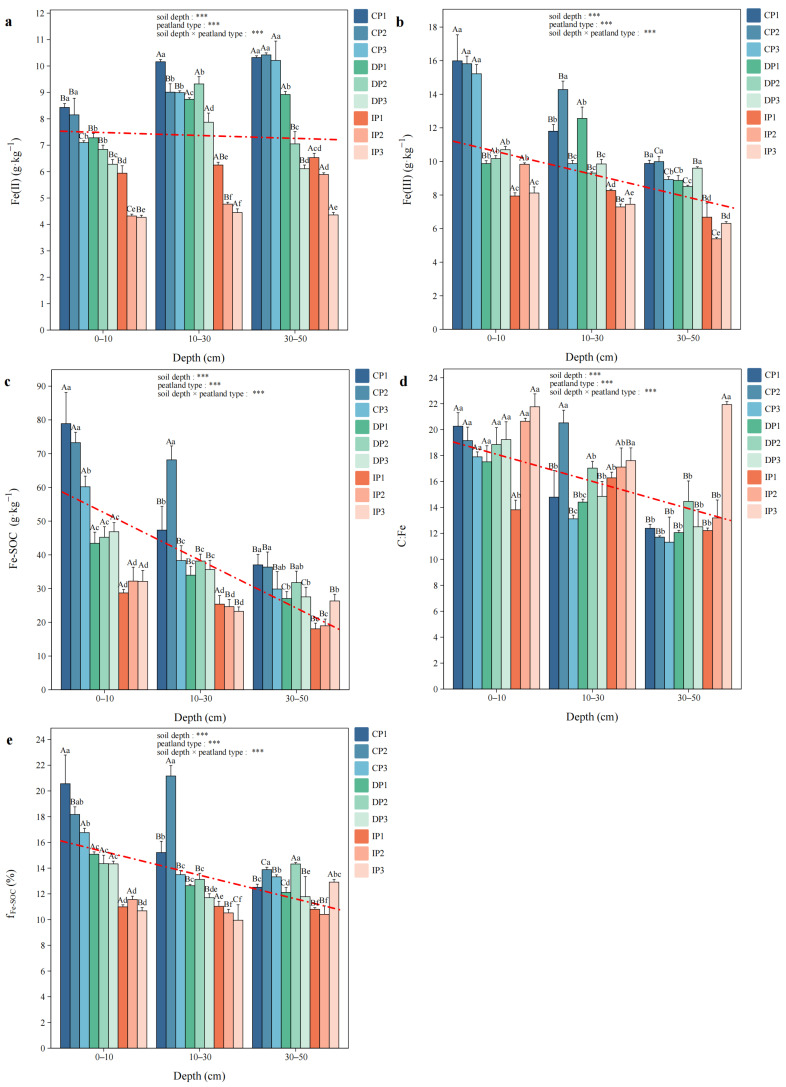
The contents of (**a**) ferrous iron (Fe(II)), (**b**) ferric iron (Fe(III)), (**c**) iron-bound soil organic carbon (Fe-SOC), (**d**) the C:Fe molar ratio, and (**e**) the proportion of Fe-SOC in total soil SOC (f_Fe-SOC_). Capital letters indicate significant differences at different soil depths in the same peatland (*p* < 0.05), and lowercase letters indicate significant differences among different peatlands at the same depth (*p* < 0.05). Two-way ANOVA was conducted to determine the effects of soil depth, peatland type, and their interactions on Fe and C coupling (*** *p* < 0.001). The red dashed lines show linear trends.

At all soil depths, Fe(II), Fe(III), Fe-SOC, and f_Fe-SOC_ generally showed a decreasing trend from CP to IP (Figure 3). The contents of Fe(II) and Fe(III) in CP were significantly higher compared to IP at all depths (*p* < 0.05). At 30–50 cm, Fe(II) content in CP was significantly higher than that at 0–10 cm (*p* < 0.05). In all peatlands, Fe(III) content was significantly lower at 30–50 cm compared to 0–10 cm (*p* < 0.05). At 0–10 cm and 10–30 cm, Fe-SOC content in IP was markedly lower compared to other permafrost zones (*p* < 0.05). The soil C:Fe molar ratio varied from 11.32 to 21.93. At 30–50 cm, IP3 showed significantly higher ratio than other peatlands (*p* < 0.05), while IP1 showed the lowest ratio at 0–10 cm. The range of f_Fe-SOC_ was from 9.95% to 21.16% in different peatlands. At 0–10 cm and 10–30 cm, the f_Fe-SOC_ in IP was significantly lower than in CP (*p* < 0.05).

### 3.5. Relationships Among Fe Phases, Soil Physicochemical Properties and Soil Enzymes

**Figure 4:**
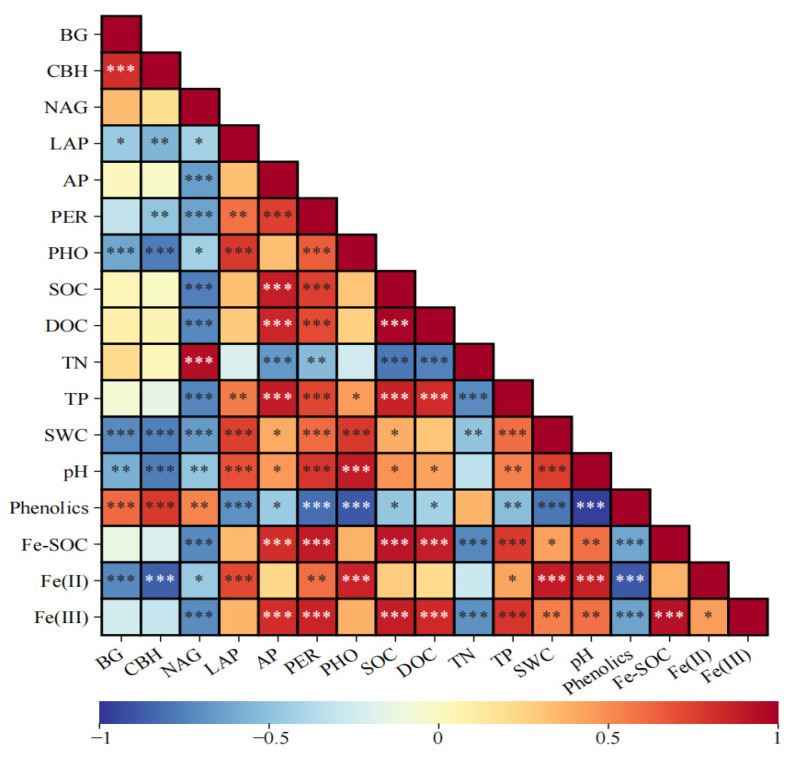
Pearson correlation analysis of soil physicochemical properties, extracellular enzyme activities, and Fe phases in permafrost peatlands of the Great Hing’an Mountains. Significance levels are indicated by asterisks: * *p* < 0.05, ** *p* < 0.01, *** *p* < 0.001.

Pearson correlation analysis showed that SOC and DOC were significantly positively related to Fe(III), Fe-SOC, pH, TP, AP, and PER activities, while showing significant negative correlations with NAG activity, TN, and phenolics (*p* < 0.05, Figure 4). BG and CBH activities were significantly negatively correlated with Fe(II), pH, SWC, PHO, and LAP activities (*p* < 0.05), while showing significant positive correlations with phenolics (*p* < 0.001). A significant positive relationship was observed between PHO activity and Fe(II), pH, SWC, LAP, and PER activities (*p* < 0.001), whereas PHO activity showed significant negative correlations with phenolics and NAG activity (*p* < 0.05). A significant positive relationship was observed between TN and NAG activity (*p* < 0.001), whereas TN showed significant negative correlations with Fe(III), Fe-SOC, SWC, TP, AP, and PER activities (*p* < 0.01). TP was significantly positively related to Fe(III), Fe(II), Fe-SOC, pH, SWC, LAP, AP, and PER activities (*p* < 0.05), while showing significant negative correlations with phenolics and NAG activity (*p* < 0.01). Notably, phenolics was significantly positively correlated with BG, CBH, and NAG activities (*p* < 0.01).

**Figure 5:**
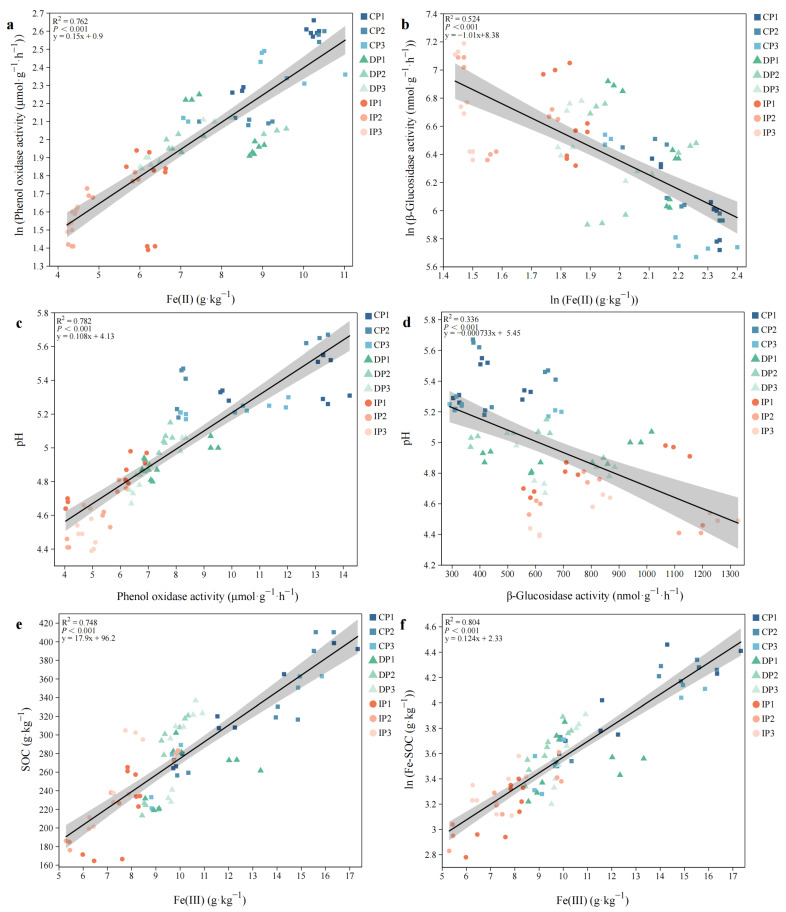
Linear regression analysis of the relationship between (**a**) Fe(II) and PHO activity, (**b**) Fe(II) and BG activity, (**c**) PHO activity and pH, (**d**) BG activity and pH, (**e**) Fe(III) and SOC, (**f**) Fe(III) and Fe-SOC.

Linear regression analysis showed that there was a highly significant positive correlation between Fe(II) and PHO activity (R^2^ = 0.762, *p* < 0.001, Figure 5a), PHO activity and pH (R^2^ = 0.782, *p* < 0.001, Figure 5c), Fe(III) and SOC (R^2^ = 0.748, *p* < 0.001, Figure 5e), as well as between Fe(III) and Fe-SOC (R^2^ = 0.815, *p* < 0.001, Figure 5f). By contrast, a negative correlation was observed between Fe(II) and BG activity (R^2^ = 0.524, *p* < 0.001, Figure 5b), BG activity and pH (R^2^ = 0.336, *p* < 0.001, Figure 5d).

**Figure 6:**
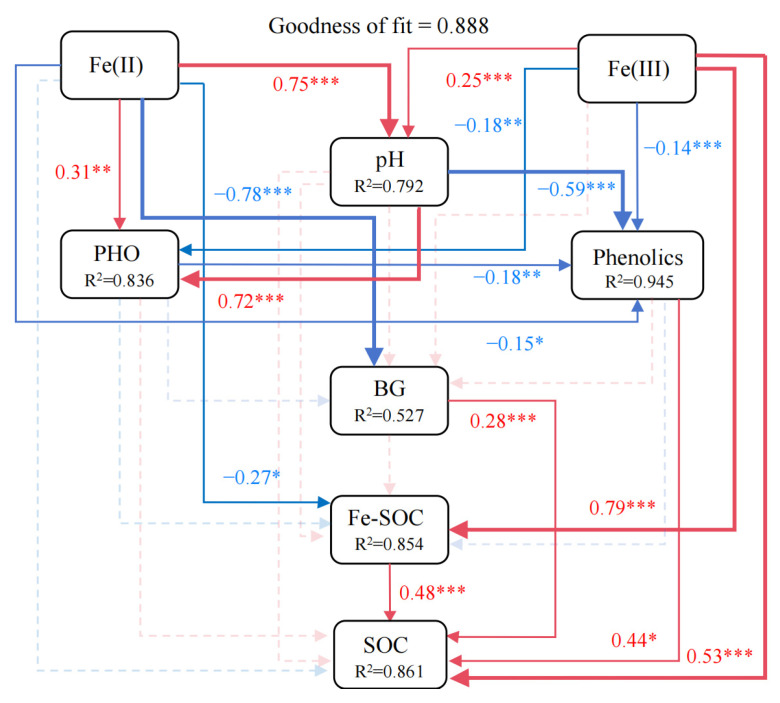
Partial least squares path modeling (PLS-PM) was used to illustrate direct and indirect influences of soil physicochemical properties, extracellular enzyme activities, and Fe phases on SOC in permafrost peatlands of the Great Hing’an Mountains. Significance levels are indicated by asterisks: * *p* < 0.05, ** *p* < 0.01, *** *p* < 0.001.

The PLS-PM analysis revealed that Fe(III), Fe-SOC, phenolics, and BG activity showed direct positive effects on SOC (coefficients = 0.53, 0.48, 0.44, and 0.28, respectively, *p* < 0.05, Figure 6). Fe(II) exhibited a significant positive association with PHO activity (*p* < 0.01). Fe(II) had no direct effect on SOC but an indirect effect on SOC via BG activity, Fe-SOC and phenolics. Fe(II) exhibited a significantly negative direct effect on Fe-SOC (coefficients = −0.27, *p* < 0.05), whereas Fe(III) exhibited a significantly positive direct effect (coefficients = 0.79, *p* < 0.001). Additionally, pH was markedly positively associated with PHO activity (*p* < 0.001). PHO activity had a significantly negative direct effect on phenolics (coefficients = −0.18, *p* < 0.01). Phenolics had no direct effect on BG activity.

## 4. Discussion

### 4.1. Enzyme Mechanisms

The accumulation of SOC in permafrost peatlands is attributed to predominant anoxic and hypoxic conditions, low temperatures, and acidic environments, all of which inhibit its solubilization and degradation through various mechanisms, including the suppression of extracellular enzyme activities [32]. Hydrolytic enzymes such as BG, CBH, NAG, LAP, and AP facilitate the breakdown of soil organic polymers, thereby meeting the carbon, nitrogen, and phosphorus requirements of both plants and microorganisms [33]. Conversely, oxidative enzymes such as PHO and PER acquire carbon and nutrients from decomposing the recalcitrant organic matter [34]. Although previous studies have reported a general lack of correlation between oxidative and hydrolytic enzyme activities [35,36], the “enzymic latch” theory proposed by Freeman et al. [8] suggests suppressed oxidative enzyme activities result in the accumulation of phenolics, thereby suppressing hydrolytic enzyme activities. However, our findings did not support a key assumption of the “enzymic latch” theory, showing a significant positive correlation between phenolics and hydrolytic enzyme activities (BG, CBH, and NAG) involved in organic carbon depolymerization. In fact, substantial research has challenged the view that phenolics inhibit soil enzymes, based on diametrically contrasting results between hydrolytic enzymes and phenolics observed in northern peatlands, forest soil, paddy soil, and alpine wetlands [9,37,38,39]. Xin et al. [40] revealed that the fate of soil phenolics, which is governed primarily by adsorption and biodegradation, is strongly influenced by soil pH. The protonation of phenolics under acidic conditions facilitates their adsorption to soil minerals or metal ions, consequently diminishing their negative impact on enzyme activity, which is consistent with our findings in different permafrost peatlands. Moreover, certain phenolics serve as accessible carbon substrates and potential signaling molecules, stimulating microbial metabolism and enhancing hydrolytic enzyme production [41].

Moreover, contrary to the “enzymic latch” theory, we found no significant positive correlation between PHO activity and hydrolytic enzyme activities, except for LAP. The reason may be that the regulation by complex biotic and abiotic factors, particularly considering their interactions, often presents a challenge to the application of these principles [42]. Our results showed a general decrease in PER and PHO activities from the continuous to the isolated permafrost zone, whereas BG, CBH, and NAG activities exhibited an opposite trend. Across different permafrost zones, the distinct responses of hydrolytic and oxidative enzymes are likely driven primarily by microbial nutrient demand for limiting nutrients and environmental factors. Soil microorganisms generally synthesize extracellular enzymes following the most economical principles [43], and our results also indicated that the activities of BG and CBH increase with the decrease in SOC and DOC contents across the permafrost gradient. However, NAG activity did not decrease despite the relief from nitrogen limitation with soil depth, suggesting that microorganisms might passively acquire a portion of nitrogen when they mineralize SOC to obtain carbon, potentially explaining why the economical principles could not fully elucidate NAG activity. Previous studies have demonstrated that most hydrolytic enzyme activities decrease with increasing soil pH [44], while neutral conditions have been shown to be more favorable for PHO activity [45], and we observed a similar result. Furthermore, the negative correlation between soil hydrolytic enzyme activities and SWC implied that the degradation of permafrost led to a decline in the water table, which may stimulate these enzyme activities, thereby accelerating carbon loss in peatlands. It should be emphasized that the enzyme activity measured at 20 °C in this study represents the potential activity under optimal conditions, which is likely higher than the actual reaction rates in situ in permafrost peatlands.

### 4.2. Preservation of SOC in Peatlands by Fe

Accumulating research has revealed that Fe governs SOC dynamics via the “iron gate” mechanism, whereby the oxidation of Fe(II) to Fe(III) inhibits PHO activity and promotes Fe-SOC association [46,47]. Supporting the “iron gate” mechanism, we observed a remarkable positive correlation between Fe(II) and PHO activity. Fe(II) may stimulate PHO activity through hydroxyl radical generation mediated by the Fenton reaction [48]. According to the “iron gate” mechanism, Fe(II) is oxidized to Fe(III), which adsorbs and co-precipitates with SOC to form Fe-SOC that protects SOC against microbial decomposition [13]. Similarly, linear regression analysis revealed that Fe(III) was positively correlated with both SOC and Fe-SOC. The content of Fe-SOC ranged from 18.07 to 78.89 g·kg^−1^, with an average of 38.10 ± 3.05 g·kg^−1^, a value higher than the averages found in forest (3–19 g·kg^−1^), sediments (0.36–14.59 g·kg^−1^) and wetland (2.0–31.2 g·kg^−1^) [31,49,50]. The range of f_Fe-SOC_ was from 9.95% to 21.16% in different peatlands, with an average of 13.46 ± 0.56% of the SOC being directly bound by Fe, which is equivalent to or lower than other ecosystems [51,52]. This finding highlighted the significance of the underappreciated Fe-SOC pool for the conservation of carbon in permafrost peatlands.

From the continuous to the isolated permafrost zone, a general decrease in Fe-SOC, f_Fe-SOC_, and Fe(III) was observed. These results indicated that the thawing of underlying permafrost destroyed the impermeable barrier in peatlands, thereby altering redox conditions and microbial activity, which could lead to the loss of accumulated Fe and the mobilization of bound organic carbon, potentially creating a positive climate feedback loop. Patzner et al. [53] also confirmed that permafrost thaw not only induced reducing conditions and promoted the proliferation of Fe(III)-reducing bacteria (e.g., *Geobacter* and *Shewanella*) but also facilitated the release of Fe and carbon, ultimately resulting in the disruption of the rusty carbon sink along the thaw gradient. Profile depth appears to be another regulator of Fe-SOC, as evidenced by significantly higher values at 0–10 cm compared to 30–50 cm in different peatlands. Facilitated by intermittent anaerobic conditions in surface layers, organic matter precipitated onto Fe oxides, likely resulting in the higher contents of Fe(III) and Fe-SOC [54]. In contrast, the long-term cooler and anoxic conditions in the deep layer favor dissimilatory Fe reduction by specialized microorganisms, which leads to the predominance of Fe(II) over Fe(III) [55]. The C:Fe molar ratio serves as an indicator of the binding mechanism between SOC and Fe, with a higher ratio suggesting greater association efficiency [56]. The molar ratio of C:Fe in different peatlands ranged from 11.32 to 21.93, with a mean of 16.17 ± 0.64, implying that co-precipitation was the main protection mechanism for SOC and possessed a strong carbon sequestration capacity in the permafrost peatlands of the Great Hing’an Mountains.

### 4.3. Factors Regulating SOC Stability in Permafrost Peatlands

The control exerted by abiotic factors on soil extracellular enzymes generally exceeds that of microbial influences [57]. According to a global meta-analysis, both soil pH and SWC serve as key determinants mediating SOC decomposition and sequestration by regulating enzyme activities [58]. Our results showed the activities of BG, CBH, and NAG were positively correlated with pH and SWC, whereas LAP, AP, PER, and PHO activities exhibited negative correlations, providing further support for the above viewpoint. Soil pH can also mediate the redox of Fe oxides and thus the complexation with SOC, whereas SWC controls the balance of SOC decomposition and stabilization by determining the contents of Fe oxides and phenolics [59,60]. Based on linear regression and PLS-PM analysis, Fe(III) was identified as a dominant factor governing SOC and Fe-SOC contents. Furthermore, the availability of nitrogen and phosphorus in soil also has an impact on SOC and DOC [61]. In isolated permafrost peatlands, higher TN content promotes plant growth and lignin production, thereby enhancing the input of phenolics, which is in agreement with our observed results [62]. Some bacteria can utilize phenolics as a carbon and energy source, driving the reductive dissolution of iron oxides and thereby mobilizing the previously protected organic carbon [63]. Conversely, phosphorus availability enhances the proportion of SOC derived from microbial necromass [64]. The adsorption of phosphorus onto organo-metallic complexes effectively suppresses Fe reduction while simultaneously stabilizing formed Fe-SOC [65]. The higher Fe-SOC content in the continuous permafrost zone could be attributed to elevated TP content when compared to the isolated permafrost zone. Temperature driven iron redox transitions may dictate the stability of Fe-SOC complexes along the permafrost gradient, preserving them in the cold continuous permafrost zone while accelerating dissolution in the warm isolated permafrost zone [66]. Permafrost degradation induces shrub expansion, which in turn affects Fe-SOC dynamics by altering vegetation type, root exudates and litter inputs [67]. Hence, permafrost thaw alters soil nutrients, environmental gradients, and hydrological regimes, which could govern the production and persistence of Fe-SOC, so projecting the future carbon fate of permafrost peatlands under climate change requires a thorough understanding of the complex underlying mechanisms behind these changes.

## 5. Conclusions

Our study systematically explored the contents, characteristics, and spatial variability of extracellular enzymes (hydrolytic and oxidative enzymes), Fe phases, and Fe-SOC in permafrost peatlands of the Great Hing’an Mountains, along with the impacts of soil physicochemical properties. Hydrolytic enzyme activities (BG, CBH, and NAG) showed positive correlations with phenolics but negative correlations with phenol oxidase (PHO) activity, which was inconsistent with the “enzymic latch” theory. However, Fe(II) showed a significant positive correlation with PHO activity, while Fe(III) protected SOC from degradation by forming Fe-SOC through co-precipitation, supporting the “iron gate” theory. This study revealed that Fe(III) served as a primary regulator determining the contents of SOC and Fe-SOC. Notably, the continuous permafrost zone had higher Fe-SOC, Fe(III), and f_Fe-SOC_ in peatlands than the isolated permafrost zone, indicating a stronger carbon sequestration capacity. Climate change drives permafrost thaw, which could weaken the protective effect of the “iron gate” mechanism in peatlands, potentially causing elevated greenhouse gas emissions as well as intensified global warming. This study provided a novel perspective for understanding the carbon protection mechanisms in permafrost peatlands, offering valuable insights for predicting the feedback effects of peatland carbon pools under future climate scenarios.

## Figures and Tables

**Table 1:** Soil physicochemical properties at various soil depths in different peatlands.

Sites	Soil Depth (cm)	SOC (g·kg^−1^)	DOC (mg·kg^−1^)	TN (g·kg^−1^)	TP (g·kg^−1^)	SWC (%)	pH	Phenolics (mg·kg^−1^)
	0–10	385.29 ± 11.60 Aa	435.85 ± 9.52 Aab	7.79 ± 0.62 Cd	3.26 ± 0.03 Abc	66.25 ± 0.27 Ca	5.32 ± 0.07 Bab	15.18 ± 0.49 Af
CP1	10–30	311.75 ± 8.29 Ba	358.16 ± 8.90 Ba	10.31 ± 0.27 Bc	3.02 ± 0.04 Ba	70.15 ± 0.69 Ba	5.53 ± 0.05 Aa	12.96 ± 0.25 Bh
	30–50	270.09 ± 12.77 Ba	316.27±8.25 Ca	12.08 ± 0.37 Ad	2.11 ± 0.04 Ca	72.36 ± 0.62 Aa	5.29 ± 0.02 Bb	15.22 ± 0.20 Ae
	0–10	403.58 ± 14.51 Aa	468.96 ± 14.07 Aa	8.87 ± 0.25 Bcd	3.01 ± 0.07 Ac	61.85 ± 1.01 Bb	5.45 ± 0.04 Aa	15.81 ± 0.79 Ae
CP2	10–30	321.88 ± 11.11 Ba	363.05 ± 10.69 Ba	10.33 ± 0.38 Bc	2.61 ± 0.05 Bb	67.44 ± 0.66 Ab	5.21 ± 0.02 Bb	15.75 ± 0.23 Ag
	30–50	262.91 ± 20.48 Ca	300.54 ± 9.63 Cab	15.94 ± 0.59 Abc	1.97 ± 0.07 Ca	70.85 ± 1.59 Aa	5.65 ± 0.09 Aa	11.33 ± 0.13 Bf
	0–10	358.74 ± 13.18 Aa	391.25 ± 10.48 Ab	11.70 ± 0.43 Bb	3.62 ± 0.08 Aa	67.39 ± 0.87 Ba	5.19 ± 0.01 Bb	17.78 ± 0.39 ABd
CP3	10–30	283.69 ± 8.36 Ba	339.61 ± 9.58 Ba	13.38 ± 0.93 ABab	3.12 ± 0.03 Ba	72.51 ± 0.72 Aa	5.26 ± 0.02 Ab	17.24 ± 0.24 Bf
	30–50	224.73 ± 12.24 Cab	268.95 ± 11.70 Cb	15.68 ± 0.55 Ac	2.27 ± 0.06 Ca	73.08 ± 1.45 Aa	5.23 ± 0.01 ABb	18.55 ± 0.16 Ad
	0–10	288.61 ± 10.77 Ac	328.74 ± 11.04 Ade	11.52 ± 1.23 Bb	2.62 ± 0.10 Ad	61.58 ± 2.11 Bb	5.02 ± 0.03 Acd	20.02 ± 0.12 Bcd
DP1	10–30	269.18 ± 9.24 Ab	302.85 ± 13.82 ABb	12.41 ± 0.44 ABa	2.71 ± 0.04 Ab	68.95 ± 0.43 Ab	4.91 ± 0.03 ABd	21.93 ± 0.32 Ad
	30–50	223.85 ± 5.24 Bab	263.21 ± 9.79 Bb	15.32 ± 0.44 Ac	1.13 ± 0.17 Bc	63.47 ± 0.36 Bb	4.83 ± 0.02 Bcd	20.17 ± 0.21 Bc
	0–10	315.49 ± 7.51 Ab	357.48 ± 13.53 Ac	7.36 ± 0.26 Cd	3.32 ± 0.03 Ab	66.85 ± 0.52 Aa	4.88 ± 0.03 Be	21.81 ± 0.57 Abc
DP2	10–30	291.05 ± 7.93 Aa	330.92 ± 13.55 Aa	10.79 ± 0.55 Bc	2.75 ± 0.05 Bb	69.01 ± 0.87 Ab	5.09 ± 0.03 Ac	21.09 ± 0.13 Ad
	30–50	221.56 ± 9.38 Bab	258.72 ± 9.32 Bb	12.88 ± 0.35 Ad	1.21 ± 0.05 Cc	63.78 ± 0.43 Bb	5.01 ± 0.02 Ac	20.23 ± 0.11 Bc
	0–10	327.25 ± 14.35 Abc	356.03 ± 11.60 Ac	10.11 ± 0.44 Bbc	2.61 ± 0.09 Ad	66.43 ± 0.37 Aa	4.82 ± 0.06 ABe	23.95 ± 1.00 Ab
DP3	10–30	304.68 ± 18.32 Aa	349.66 ± 7.22 Aa	11.35 ± 0.15 Bbc	2.06 ± 0.07 Bc	63.37 ± 0.73 Bc	5.03 ± 0.02 Ac	20.76 ± 0.22 Be
	30–50	233.67 ± 15.30 Ba	285.19 ± 12.51 Ba	14.26 ± 0.38 Acd	1.58 ± 0.06 Cb	62.17 ± 0.67 Bb	4.72 ± 0.09 Bd	24.62 ± 0.28 Ab
	0–10	261.28 ± 6.77 Ad	310.52 ± 7.70 Ae	16.25 ± 1.01 Aa	2.03 ± 0.03 Ae	53.74 ± 0.49 Bc	4.95 ± 0.03 Ade	22.87 ± 0.21 Bb
IP1	10–30	230.37 ± 11.15 Ac	285.76 ± 6.78 Acd	13.75 ± 0.18 Ba	1.25 ± 0.04 Be	58.62 ± 0.16 Ad	4.67 ± 0.03 Ce	24.05 ± 0.42 Ac
	30–50	167.55 ± 9.60 Bbc	208.15 ± 12.10 Be	18.37 ± 0.57 Aa	1.06 ± 0.03 Ccd	55.62 ± 0.84 Bc	4.82 ± 0.02 Bcd	24.19 ± 0.24 Ab
	0–10	278.64 ± 17.71 Ad	332.08 ± 8.26 Ad	15.81 ± 0.76 Ba	2.21 ± 0.04 Ae	51.85 ± 0.66 Bc	4.43 ± 0.02 Cf	27.27 ± 0.79 Aa
IP2	10–30	233.97 ± 11.19 ABc	289.54 ± 11.64 Bc	12.27 ± 0.41 Babc	1.62 ± 0.05 Bd	56.13 ± 0.89 Ad	4.58 ± 0.04 Be	27.09 ± 0.13 Ab
	30–50	182.43 ± 9.91 Bb	231.15 ± 8.56 Cde	18.85 ± 1.31 Aa	0.98 ± 0.03 Ccd	50.52 ± 0.87 Bd	4.77 ± 0.03 Ad	24.20 ± 0.25 Bb
	0–10	300.82 ± 8.75 Ac	375.86 ± 15.38 Ac	14.42 ± 0.63 Ba	2.15 ± 0.09 Ae	47.55 ± 1.45 Bd	4.51 ± 0.02 Bf	27.79 ± 0.17 ABa
IP3	10–30	223.95 ± 10.51 Bc	274.65 ± 6.79 Bd	11.39 ± 0.31 Cbc	1.36 ± 0.10 Be	52.38 ± 0.66 Ae	4.40 ± 0.02 Cf	28.38 ± 0.20 Aa
	30–50	203.81 ± 11.59 Bbc	248.97 ± 9.90 Bc	18.06 ± 0.67 Aa	0.79 ± 0.06 Cd	45.19 ± 0.49 Be	4.63 ± 0.03 Ad	27.22 ± 0.22 Ba

Note: Data are presented as mean ± standard error (*n* = 3). Capital letters indicate significant differences at different soil depths in the same peatland (*p* < 0.05), and lowercase letters indicate significant differences among different peatlands at the same depth (*p* < 0.05). CP, DP, and IP refer to the continuous, discontinuous, and isolated permafrost zones, respectively.

## Data Availability

The data presented in this study are available within the article.
